# Combined Effects of Puerarin and Adipose‐Derived Stem Cells on Alveolar Bone Preservation and Inflammation Control in Periodontitis Through p38MAPK Modulation

**DOI:** 10.1002/kjm2.70182

**Published:** 2026-02-03

**Authors:** Ting Yang, Xu Zhang, Liang‐Fu Zhang

**Affiliations:** ^1^ Department of Emergency and General Dentistry Changsha Stomatological Hospital Changsha Hunan China

**Keywords:** adipose‐derived stem cells, inflammation, p38MAPK signaling, periodontitis, Puerarin

## Abstract

This study evaluated the effects of puerarin and adipose‐derived stem cells (ADSCs), alone or combined, on p38MAPK activity, alveolar bone preservation, and inflammatory responses in a rat periodontitis (PD) model and in vitro. ADSCs were exposed to various puerarin concentrations to assess cell proliferation, osteogenic differentiation, and p38MAPK‐related protein expression. Additional experiments employed anisomycin (a p38 MAPK activator) and *Porphyromonas gingivalis* LPS (Pg‐LPS) to determine whether puerarin attenuates p38MAPK overactivation and reduces pro‐inflammatory cytokine production. In vivo, ligature‐ and *Porphyromonas gingivalis* –induced periodontitis rats were randomized to Normal, PD, PD + ADSCs, PD + puerarin, or PD + puerarin + ADSCs groups, and alveolar bone microarchitecture (micro‐CT) and periodontal p38MAPK activation and osteogenic/inflammatory proteins (Western blot, ELISA) were assessed. At 10^−6^ M, puerarin significantly increased ADSC proliferation and osteogenic differentiation, whereas anisomycin activation diminished these benefits, which were restored by co‐treatment with puerarin. Puerarin also reduced Pg‐LPS induced secretion of pro‐inflammatory cytokines by suppressing p38MAPK. In vivo, the periodontitis group showed substantial alveolar bone loss and marked inflammatory cell infiltration. Both ADSCs and puerarin partially alleviated these changes but did not fully reverse them. Notably, their combination provided the greatest benefit, nearly normalizing alveolar bone parameters, strongly inhibiting p38MAPK, and further reducing inflammatory cytokine levels in periodontal tissues. Collectively, puerarin and ADSCs exert complementary anti‐inflammatory and pro‐osteogenic effects associated with attenuation of p38MAPK signaling. Co‐administration produced superior therapeutic outcomes, supporting this dual approach as a potential strategy for periodontitis‐associated bone loss.

## 1 Introduction

Periodontitis is a chronic inflammatory disease characterized by progressive destruction of periodontal tissues, affecting billions of individuals globally [1]. Primarily driven by polymicrobial dysbiosis and exacerbated by dysregulated host immune responses, periodontitis results in persistent inflammation, connective tissue breakdown, and alveolar bone loss, ultimately leading to tooth loss if untreated [2, 3]. Effective therapeutic approaches targeting both inflammatory control and tissue regeneration remain critical yet challenging in clinical periodontal management.

Puerarin, also known as daidzein‐8‐C‐glucoside, is a major bioactive isoflavone isolated from the root of kudzu ( *Pueraria lobata* ), serving as a chemotaxonomic marker and responsible for many pharmacological activities attributed to the genus Pueraria [4]. Due to its phytoestrogenic and anti‐inflammatory properties, puerarin has shown therapeutic promise in various inflammatory and bone‐related diseases. Previous studies demonstrated that puerarin effectively suppresses receptor activator of NF‐κB ligand (RANKL)‐induced osteoclastogenesis by inhibiting CREB/PGC1β/c‐Fos/NFATC1 signaling pathways, suggesting potential applications in diseases like osteoporosis, rheumatoid arthritis, and periodontitis [5]. Furthermore, puerarin inhibits lipopolysaccharide (LPS)‐induced osteoclast differentiation and bone resorption through suppression of Akt signaling pathways, reinforcing its therapeutic potential against bacterially induced bone diseases, including periodontitis [6].

Puerarin exerts significant physiological effects on various stem cell populations, including their self‐renewal/proliferation, differentiation and apoptosis [7]. It has been shown to enhance osteogenic differentiation and proliferation of human periodontal ligament stem cells (PDLSCs), further supporting its possible use in periodontal regeneration [8]. Adipose‐derived stem cells (ADSCs) represent a promising cell source for tissue regeneration due to their accessibility, multipotency, immunomodulatory capabilities, and robust osteogenic differentiation potential [9]. Recent studies have increasingly focused on the use of ADSCs in periodontal tissue engineering, highlighting their beneficial role in attenuating inflammation and promoting periodontal regeneration through osteogenic differentiation and secretion of anti‐inflammatory factors [10, 11]. Despite considerable interest, strategies that further potentiate ADSC‐mediated therapeutic effects in periodontitis remain an area of ongoing research, particularly involving combinations with bioactive compounds such as puerarin.

The p38 mitogen‐activated protein kinase (p38MAPK) signaling pathway is well established as a critical mediator in inflammatory responses and bone metabolism associated with periodontal diseases [12]. Aberrant activation of p38MAPK enhances pro‐inflammatory cytokine production, aggravating periodontal inflammation, tissue destruction, and alveolar bone loss [13]. Notably, puerarin has recently been reported to delay mammary gland aging by effectively inhibiting p38MAPK signaling [14], suggesting a broader potential to modulate inflammation‐related pathways. Thus, targeting the p38MAPK pathway may offer a promising strategy for periodontal inflammation control and tissue regeneration.

Given the individual regenerative and anti‐inflammatory potentials of ADSCs and puerarin, we hypothesize that their combination may yield synergistic effects in managing periodontitis by simultaneously inhibiting inflammation via p38MAPK pathway modulation and enhancing osteogenic differentiation. To date, however, the specific mechanisms underlying this potential synergy remain poorly understood. Therefore, the present study aimed to evaluate the combined therapeutic effects of puerarin and ADSCs in a rat periodontitis model and associated in vitro assays, with particular focus on their influence on alveolar bone preservation, inflammation control, and regulation of p38MAPK signaling and osteogenic differentiation.

## 2 Materials and Methods

### 2.1 Experimental Animals

This study was conducted using Sprague–Dawley (SD) rats, and all experimental procedures were performed in accordance with established ethical standards for animal experimentation [15] and approved by the ethical committee of our hospital (HNUCN11‐2409‐320). Thirty male Sprague–Dawley rats (9–11 weeks old) were purchased from Shanghai SLAC Laboratory Animal Co. Ltd. (Shanghai, China). Throughout the experiment, animals were housed in a standard environment (temperature 21°C ± 1°C, humidity 50%–55%, 12‐h light/dark cycle), with free access to water and standard feed. All rats were acclimatized for 1 week before the start of the experiment.

### 2.2 Identification of Tri‐Lineage Differentiation Potential of ADSCs

StemPro ADSCs (Catalog No. R7788115, Thermo Fisher Scientific, USA), originating from human adipose tissue through enzymatic digestion, were cultured as low‐passage (P1) adherent cells. The StemPro series of differentiation media was used to evaluate the adipogenic, chondrogenic, and osteogenic potentials of ADSCs. For adipogenesis, hADSCs were seeded at a density of 1 × 10^4^ cells/cm^2^ for adipogenic differentiation; after cell attachment, the medium was switched to adipogenic differentiation medium for 14 days, followed by Oil Red O staining to detect lipid droplets. For chondrogenesis, cells were seeded at the same density and cultured until 60%–80% confluency, then transferred to chondrogenic induction medium for 21 days, after which Alcian Blue staining was performed to assess glycosaminoglycan deposition. For osteogenesis, cells were seeded at 5 × 10^3^ cells/cm^2^; once they reached 60%–80% confluency, the medium was changed to an osteogenic induction medium. Alkaline phosphatase (ALP) activity (ALP Activity Kit, Catalog No. EEA002, Thermo Fisher Scientific) and osteogenic gene expression were evaluated on days 7 and 14. On day 21, Alizarin Red S staining was conducted to confirm mineralized nodule formation.

### 2.3 Cell Culture and Experimental Grouping

Initially, various concentrations of puerarin (0 M, 10^−3^ M, 10^−4^ M, 10^−5^ M, 10^−6^ M, 10^−7^ M, 10^−8^ M, Catalog No.: HY‐N0145, MedChemExpress, China) were tested to determine their effects on ADSC proliferation using MTT assay, osteogenic differentiation, and p38MAPK signaling pathway. Based on these findings, an optimal concentration was chosen for subsequent experiments.

Next, ADSCs were allocated into three groups to test whether anisomycin‐induced p38MAPK activation counteracts puerarin‐induced osteogenic effects: Control (0.1% DMSO), anisomycin (25 μg/mL; MedChemExpress, HY‐18982; 30 min), and puerarin + anisomycin. In the puerarin + anisomycin group, puerarin (10^−6^ M; DMSO ≤ 0.1%) was applied for 1 h (pretreatment), followed by anisomycin (25 μg/mL) for 30 min to activate p38MAPK [16, 17]. Cells were then lysed immediately for Western blot of p‐p38 and total p38, and the p‐p38/p38 ratio was quantified. For functional assays, cultures were continued in drug‐free growth medium (proliferation by MTT on days 1, 3, and 5) or switched to drug‐free StemPro Osteogenic Induction Medium (ALP activity and osteogenic gene expression on days 7 and 14; Alizarin Red S staining on day 21). Vehicle and anisomycin groups underwent identical medium exchanges and timing, with DMSO matched to ≤ 0.1%.

For inflammatory assays, ADSCs were assigned to four groups: control group, Pg‐LPS group (1 μg/mL *Porphyromonas gingivalis* LPS; Sigma‐Aldrich, Cat. No. SMB00610 for 24 h) [18, 19], Puerarin group (treated with 10^−6^ M puerarin for 24 h), and Puerarin + Pg‐LPS group (pre‐treated with 10^−6^ M puerarin for 1 h before co‐incubation with 1 μg/mL Pg‐LPS for 24 h). Culture supernatants were collected for ELISA to measure pro‐inflammatory cytokines. Simultaneously, total protein was extracted for Western blot analysis to evaluate p38MAPK pathway‐associated proteins. All in vitro assays were repeated in three independent experiments unless otherwise specified; notably, the key in vitro Western blot experiments were performed using six independent biological replicates.

### 2.4 MTT Cell Viability Assay

For proliferation studies, ADSCs were seeded at 5 × 10^3^ cells per well in 96‐well plates. After 24 h of adherence, the medium was replaced with fresh phenol red‐free medium containing different concentrations of puerarin, and cells were further incubated for 1, 3, or 5 days. At each time point, 10 μL of MTT stock solution (final concentration 1.2 mM) was added to each well and incubated at 37°C for 4 h. Subsequently, 100 μL of SDS‐HCl solution was added, and cells were further incubated at 37°C for 12 h to fully dissolve the formazan crystals. Optical density (OD) was measured at 570 nm in a plate reader. All experiments were conducted in triplicate wells per group, and each experiment was repeated three times.

### 2.5 One‐Step RT‐qPCR for Gene Expression

A one‐step RT‐qPCR protocol was employed to measure mRNA levels of osteogenic genes (ALP, SP7, BSP, and OCN). After cell treatments, total RNA was extracted using TRIzol reagent, and concentration/purity were determined by spectrophotometry. One microgram of total RNA served as the template for a one‐step RT‐qPCR kit. The reaction mixture (20 μL total volume) was placed in a quantitative PCR instrument with the following cycling conditions: reverse transcription at 50°C for 10 min, pre‐denaturation at 95°C for 2 min, followed by 40 cycles of 95°C for 15 s and 60°C for 30 s (annealing/extension). Glyceraldehyde‐3‐phosphate dehydrogenase (GAPDH) was used as the internal reference, and relative gene expression was calculated by the 2^‐ΔΔCt^ method. Each sample was analyzed in triplicate wells, and experiments were repeated three times.

### 2.6 Animal Model Establishment and Grouping

**FIGURE 1:**
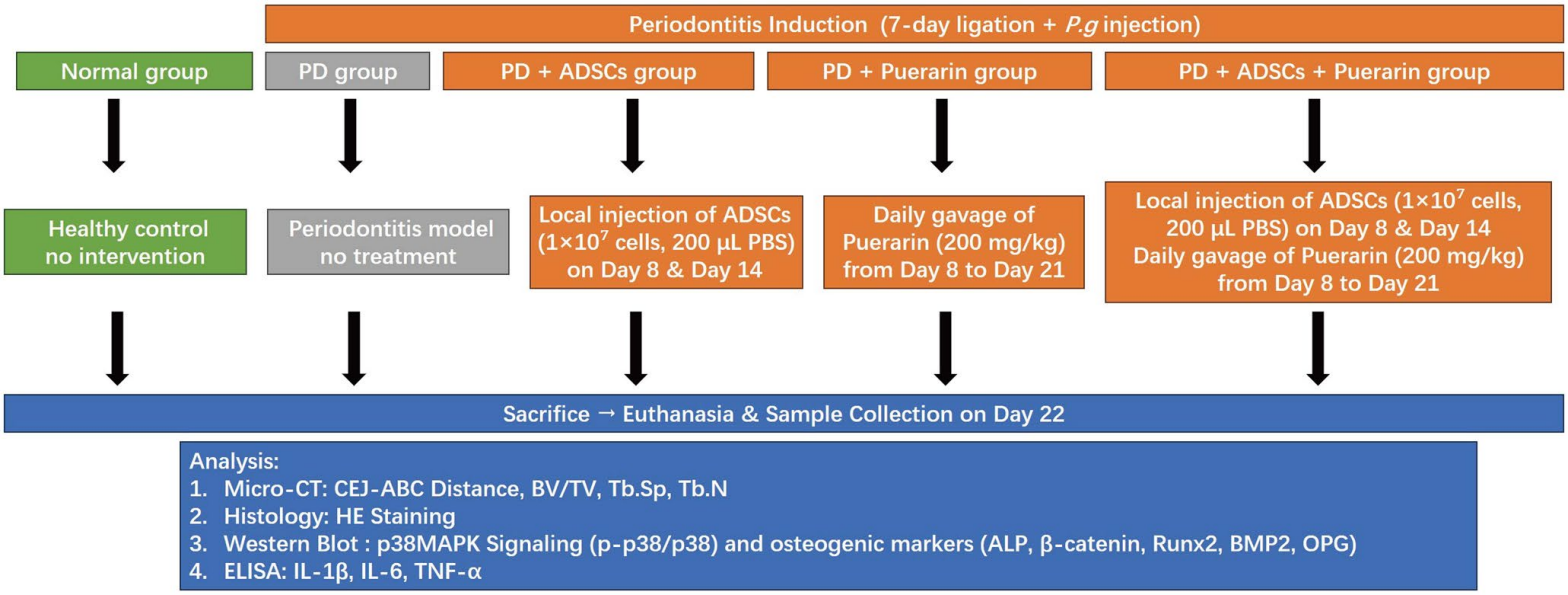
An overview of the animal experimental design.

An experimental periodontitis model was induced by placing 4–0 silk ligatures at the cervical region of the bilateral maxillary second molars and injecting 100 μL Pg suspension (5 × 10^8^ CFU/mL) into the subgingival area for 7 consecutive days [20] to establish a stable periodontitis model. Rats were randomly assigned to five groups (*n* = 6 per group; total *n* = 30): (1) Normal (no ligature or Pg; healthy control); (2) PD (ligature + Pg, no treatment); (3) PD + ADSCs; (4) PD + puerarin; and (5) PD + puerarin + ADSCs. In the PD + ADSCs group, 1 × 10^7^ ADSCs (in 200 μL PBS) were locally injected into the periodontal pockets on days 8 and 14 [21]. In the PD + puerarin groups, puerarin (200 mg/kg) was administered by daily oral gavage from day 8 to day 21 [22]; the combination group received both regimens on the same schedule. All animals were euthanized on day 22 under anesthesia, and the maxillae and periodontal tissues were collected. Maxillae were fixed in 4% paraformaldehyde for 24 h and scanned ex vivo by micro‐CT (VNC‐102, Pingsheng Medical Technology; 90 kV, 0.08 mA; FDK reconstruction). Buccal CEJ–ABC distances were measured on 3D reconstructions at predefined sites, and trabecular parameters (BV/TV, Tb.N, Tb.Sp) were quantified within a standardized cylindrical VOI (2.4‐mm diameter × 1.5‐mm height) anchored at the second molar furcation with roots excluded. Paraffin sections were prepared for hematoxylin and eosin (HE) staining. To align imaging, histology, and biochemistry, periodontal soft tissues adjacent to the maxillary second molars were harvested under the same anatomic frame for protein and cytokine assays. All dissections were performed by a single, blinded operator at the same time of day. A schematic of the experimental design was provided in Figure 1.

### 2.7 Enzyme‐Linked Immunosorbent Assay (ELISA)

For in vitro assays, culture supernatants from ADSCs were collected, and interleukin‐1β (IL‐1β), interleukin‐6 (IL‐6), and tumor necrosis factor‐α (TNF‐α) were quantified with ELISA kits (Sigma‐Aldrich). Specifically, IL‐1β was measured using kit RAB0273 (inter‐assay CV < 12%, intra‐assay CV < 10%, sensitivity 0.3 pg/mL, standard curve range 0.48–100 pg/mL), IL‐6 with kit EZIL6 (inter‐assay CV < 15%, intra‐assay CV < 5%, standard curve range 1.17–300 pg/mL, accuracy 91%–109%), and TNF‐α with kit EZHTNFA‐150 K (inter‐assay CV ≤ 7%, intra‐assay CV ≤ 3%, sensitivity 1.65 pg/mL, standard curve range 1.65–400 pg/mL). For in vivo assays, freshly collected periodontal tissues were snap‐frozen, homogenized, and assayed for IL‐1β (RAB0278; inter‐assay CV < 12%, intra‐assay CV < 10%, sensitivity 80 pg/mL, standard curve range 68.59–50,000 pg/mL), IL‐6 (RAB0312; inter‐assay CV < 12%, intra‐assay CV < 10%, sensitivity 15 pg/mL, standard curve range 40.96–10,000 pg/mL), and TNF‐α (RAB0480; inter‐assay CV < 12%, intra‐assay CV < 10%, sensitivity 25 pg/mL, standard curve range 82.3–20,000 pg/mL) according to the manufacturer's instructions.

### 2.8 Western Blot Analysis

Under a stereomicroscope, periodontal soft tissues adjacent to the buccal surface of the maxillary second molar were micro‐dissected. A narrow band of tissue extending from the gingival margin to the coronal third of the root was excised with microsurgical scissors. To prevent contamination by non‐target structures, the oral keratinized epithelium and all mineralized components (enamel, dentin, cementum, and alveolar bone) were meticulously avoided. Specimens were immediately rinsed in ice‐cold PBS, gently blotted, weighed, snap‐frozen in liquid nitrogen within 2 min, and stored at −80°C until lysis. Total protein from both cell and animal tissue samples was extracted using RIPA lysis buffer, and concentrations were determined by the bicinchoninic acid (BCA) method. Equal amounts of protein were separated by SDS‐PAGE and transferred to PVDF membranes. After blocking, membranes were incubated overnight at 4°C with primary antibodies, followed by HRP‐conjugated secondary antibodies for 1 h at room temperature. Chemiluminescence signals were developed using enhanced chemiluminescence (ECL) and imaged on a gel documentation system. Band intensities were quantified in ImageJ and normalized to a loading control (GAPDH) to obtain relative expression levels.

### 2.9 Statistical Analyses

Statistical analyses were performed using GraphPad Prism software (Version 8.0). Data are presented as mean ± standard deviation (SD). Comparisons among multiple groups at each time point were conducted using one‐way ANOVA followed by Holm‐Sidak's multiple comparisons test to adjust for multiple testing. For assessing the effects over time across different treatment groups, two‐way ANOVA was utilized, followed by Dunnett's multiple comparisons test when appropriate. Differences were considered statistically significant at *p* < 0.05.

## 3 Results

### 3.1 Morphological Characteristics and Identification of ADSCs

Flow cytometry analysis demonstrated that the ADSCs exhibited high expression (> 95%) of mesenchymal stem cell surface markers, including CD29, CD44, CD73, CD90, CD105, and CD166, while hematopoietic and endothelial markers (CD14, CD31, CD45, and Lin1) were expressed at minimal levels (< 2%) (data not shown). Following 14 days of adipogenic induction, Oil Red O staining confirmed the formation of intracellular lipid droplets. After 21 days of chondrogenic induction, Alcian Blue staining revealed a significant increase in glycosaminoglycan deposition in the extracellular matrix. Similarly, after 21 days of osteogenic induction, Alizarin Red S staining indicated the presence of mineralized nodules. These results confirm the multipotent differentiation potential of ADSCs toward adipogenic, chondrogenic, and osteogenic lineages (Supplementary Figure S1).

### 3.2 Puerarin Promoted ADSC Proliferation in a Concentration‐Dependent Manner

**FIGURE 2:**
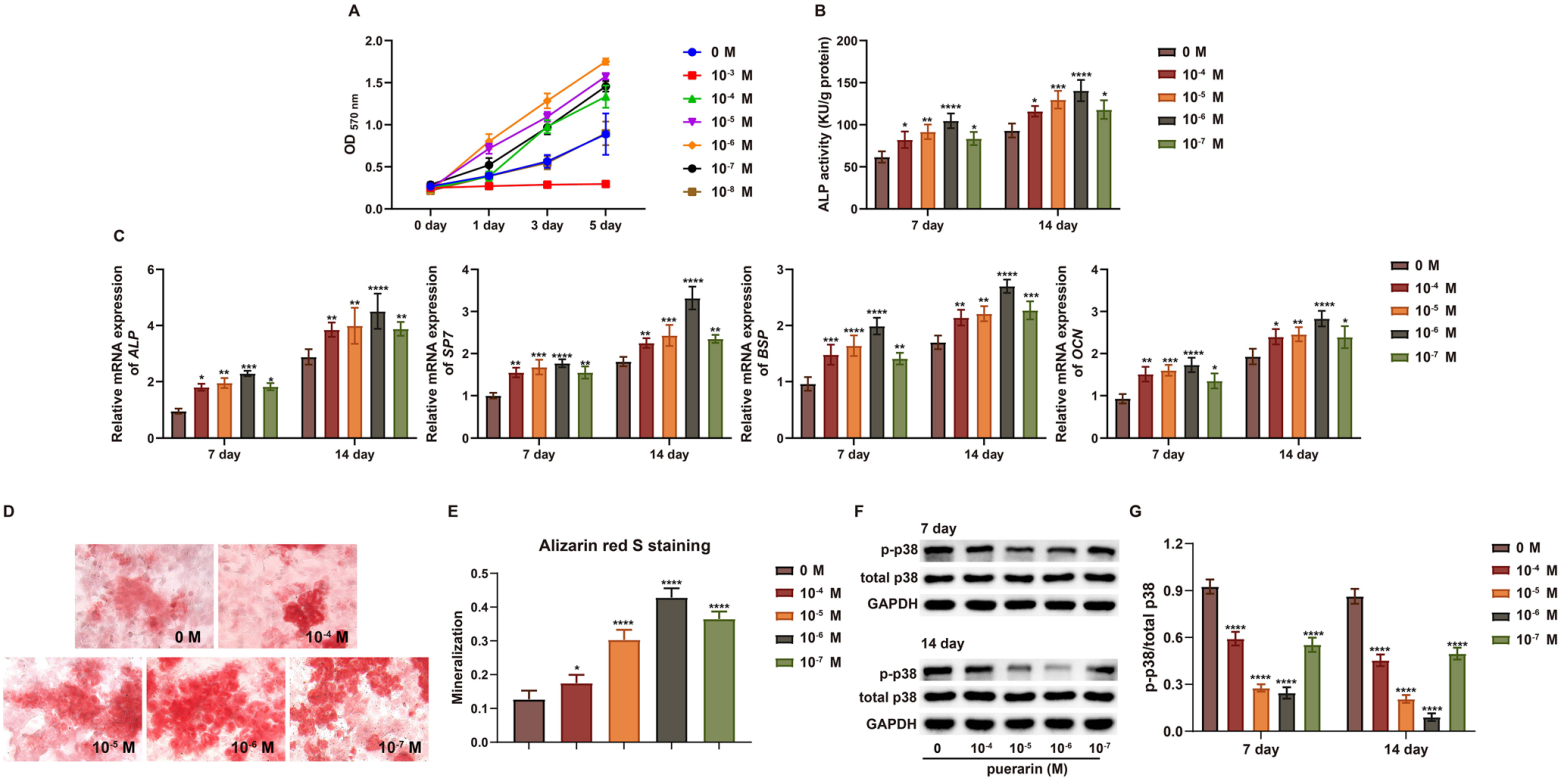
Effects of puerarin on ADSC proliferation, osteogenic differentiation, and p38MAPK signaling. *Note:* (A) MTT assay showing the proliferation of adipose‐derived stem cells (ADSCs) treated with different concentrations of puerarin at days 0, 1, 3, and 5. (B) Alkaline phosphatase (ALP) activity assay at days 7 and 14 of osteogenic induction in puerarin‐treated ADSCs. (C) Relative mRNA expression levels of osteogenic markers, including ALP, Sp7 transcription factor (SP7), bone sialoprotein (BSP), and osteocalcin (OCN), determined by quantitative real‐time polymerase chain reaction (RT‐qPCR) at days 7 and 14. (D) Alizarin Red S staining of ADSCs at day 21 of osteogenic differentiation, showing mineralized extracellular matrix deposition. (E) Quantification of Alizarin Red S staining in puerarin‐treated groups. (F,G) Western blot analysis of phosphorylated p38 (p‐p38) and total p38 expression in ADSCs at days 7 and 14 under osteogenic conditions. Data are presented as mean ± SD. Western blotting was performed with *n* = 6 independent biological replicates, whereas other in vitro assays were repeated in three independent experiments. Compared to the 0 M group: *p < 0.05, **p < 0.01, ***p < 0.001, ****p < 0.0001.

The effect of puerarin at different concentrations (0 M, 10^−3^ M, 10^−4^ M, 10^−5^ M, 10^−6^ M, 10^−7^ M, and 10^−8^ M) on ADSC proliferation was evaluated using the MTT assay (Figure 2A). On day 0, no significant differences in ADSC proliferation were observed among groups (all *p* > 0.05). On day 1, ADSC proliferation was significantly increased in the 10^−5^ M and 10^−6^ M puerarin groups compared to the control group (*p* < 0.05), whereas other groups showed no significant difference (*p* > 0.05). By day 3, ADSC proliferation was markedly enhanced in the 10^−4^ M, 10^−5^ M, 10^−6^ M, and 10^−7^ M groups (*p* < 0.05), with the greatest proliferation observed at 10^−6^ M puerarin. In contrast, the 10^−3^ M group exhibited cytotoxic effects, significantly inhibiting ADSC proliferation (*p* < 0.001). By day 5, this proliferative trend was further enhanced, with the strongest effect noted in the 10^−6^ M group, followed by the 10^−5^ M, 10^−7^ M, and 10^−4^ M groups, respectively (*p* < 0.05). Based on these results, puerarin concentrations of 10^−4^ M, 10^−5^ M, 10^−6^ M, and 10^−7^ M were selected for subsequent analyses.

### 3.3 Puerarin Enhanced Osteogenic Differentiation of ADSCs and Suppressed p38MAPK Signaling in a Concentration‐Dependent Manner

To evaluate the osteogenic effects of puerarin, ALP activity was measured on days 7 and 14 post‐induction (Figure 2B). Compared to the control (0 M), puerarin treatment significantly increased ALP activity in a concentration‐dependent manner (all *p* < 0.05), with the highest levels observed in the 10^−6^ M group (*p* < 0.0001). Similarly, RT‐qPCR analysis demonstrated a significant upregulation of osteogenic genes (ALP, SP7, BSP, OCN) at both time points (all *p* < 0.05), with the most pronounced effect in the 10^−6^ M group (all *p* < 0.001, Figure 2C). After 21 days of induction, Alizarin Red S staining confirmed enhanced extracellular matrix mineralization in all puerarin‐treated groups (all *p* < 0.05), with the greatest mineral deposition in the 10^−6^ M group (*p* < 0.01, Figure 2D,E). Western blot analysis further revealed that puerarin significantly inhibited p38 phosphorylation (p‐p38/p38 ratio) at both time points (all *p* < 0.001), with the strongest suppression observed in the 10^−5^ M and 10^−6^ M groups (Figure 2F,G). Given that 10^−6^ M puerarin exhibited the most potent effects on ADSC proliferation, osteogenic differentiation, and p38MAPK inhibition, these findings suggest that puerarin enhances osteogenic differentiation by suppressing p38MAPK signaling.

### 3.4 Puerarin Restored ADSC Proliferation and Osteogenic Differentiation Suppressed by Anisomycin

**FIGURE 3:**
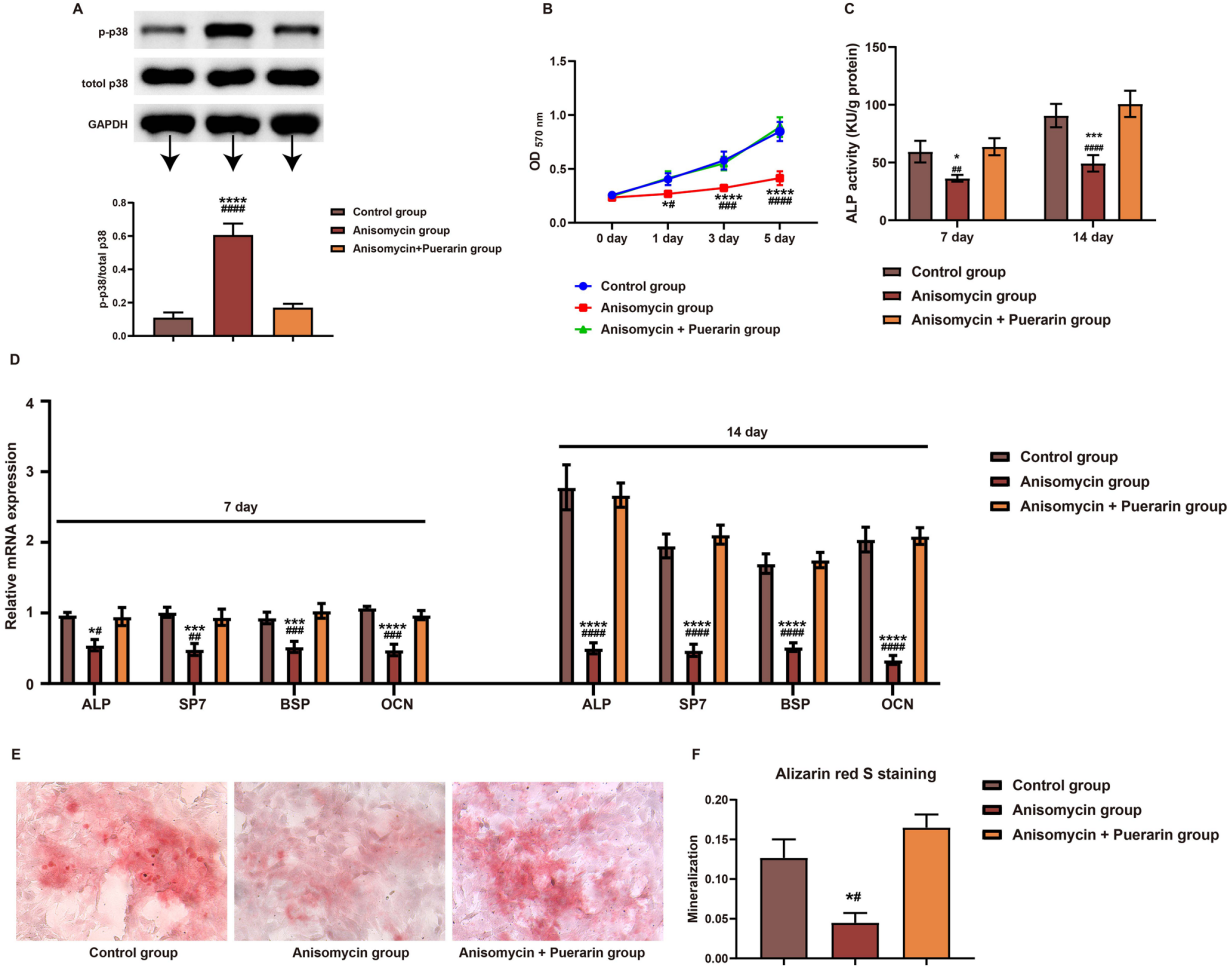
Puerarin attenuated anisomycin‐induced p38MAPK activation and rescued ADSC proliferation and osteogenic differentiation. *Note:* (A) Representative Western blot and densitometric analysis of p‐p38 and total p38 in ADSCs treated with anisomycin with or without puerarin; GAPDH served as a loading control. (B) MTT assay measuring ADSC proliferation in the control, anisomycin, and anisomycin + puerarin groups at days 0, 1, 3, and 5. (C) ALP activity assay at days 7 and 14 in each group. (D) RT‐qPCR analysis of osteogenic gene expression (ALP, SP7, BSP, OCN) in ADSCs at days 7 and 14. (E) Alizarin Red S staining of ADSCs after 21 days of osteogenic induction. (F) Quantification of Alizarin Red S staining. Data are presented as mean ± SD. Western blotting was performed with *n* = 6 independent biological replicates, whereas other in vitro assays were repeated in three independent experiments. Compared to the control group: *p < 0.05, **p < 0.01, ***p < 0.001, ****p < 0.0001. Compared to the anisomycin + puerarin group: #*p* < 0.05, ##*p* < 0.01, ###*p* < 0.001, ####*p* < 0.0001.

To determine whether p38MAPK activation contributes to the inhibitory effects on ADSC osteogenesis, anisomycin was applied to activate p38MAPK, and puerarin was co‐administered to evaluate its protective effects. As shown in Figure 3A, anisomycin markedly increased p38 phosphorylation (p‐p38/p38 ratio) compared with the control group (*p* < 0.05), whereas puerarin co‐treatment significantly reduced anisomycin‐induced p38 phosphorylation, bringing the p‐p38/p38 ratio close to the control level (*p* < 0.05). Consistently, anisomycin significantly suppressed ADSC proliferation, as indicated by lower OD values in the MTT assay at **days 1, 3, and 5** versus the control group (Figure 3B, all *p* < 0.05). In contrast, co‐treatment with puerarin substantially restored proliferation, with OD values approaching those of the control group by day 5 (*p* > 0.05). During osteogenic induction, anisomycin significantly reduced ALP activity at days 7 and 14 (all *p* < 0.05, Figure 3C). This was accompanied by marked downregulation of osteogenic marker mRNAs (ALP, SP7, BSP, and OCN) at both time points (Figure 3D, all *p* < 0.05). Notably, puerarin co‐treatment effectively reversed these inhibitory effects, restoring ALP activity and osteogenic gene expression to levels comparable to the control group (*p* > 0.05). After 21 days of osteogenic induction, Alizarin Red S staining demonstrated a pronounced reduction in mineralized nodule formation in the anisomycin group (Figure 3E), which was confirmed by quantitative analysis (Figure 3F *p* < 0.05 vs. control). In the anisomycin + puerarin group, mineral deposition was significantly increased compared with anisomycin alone (*p* < 0.05) and was comparable to the control group (*p* > 0.05). Collectively, these data indicate that puerarin mitigates anisomycin‐induced p38MAPK activation and rescues ADSC proliferation and osteogenic differentiation.

### 3.5 Puerarin Attenuated Pg‐LPS Induced Inflammatory Cytokine Release and p38MAPK Activation in ADSCs

**FIGURE 4:**
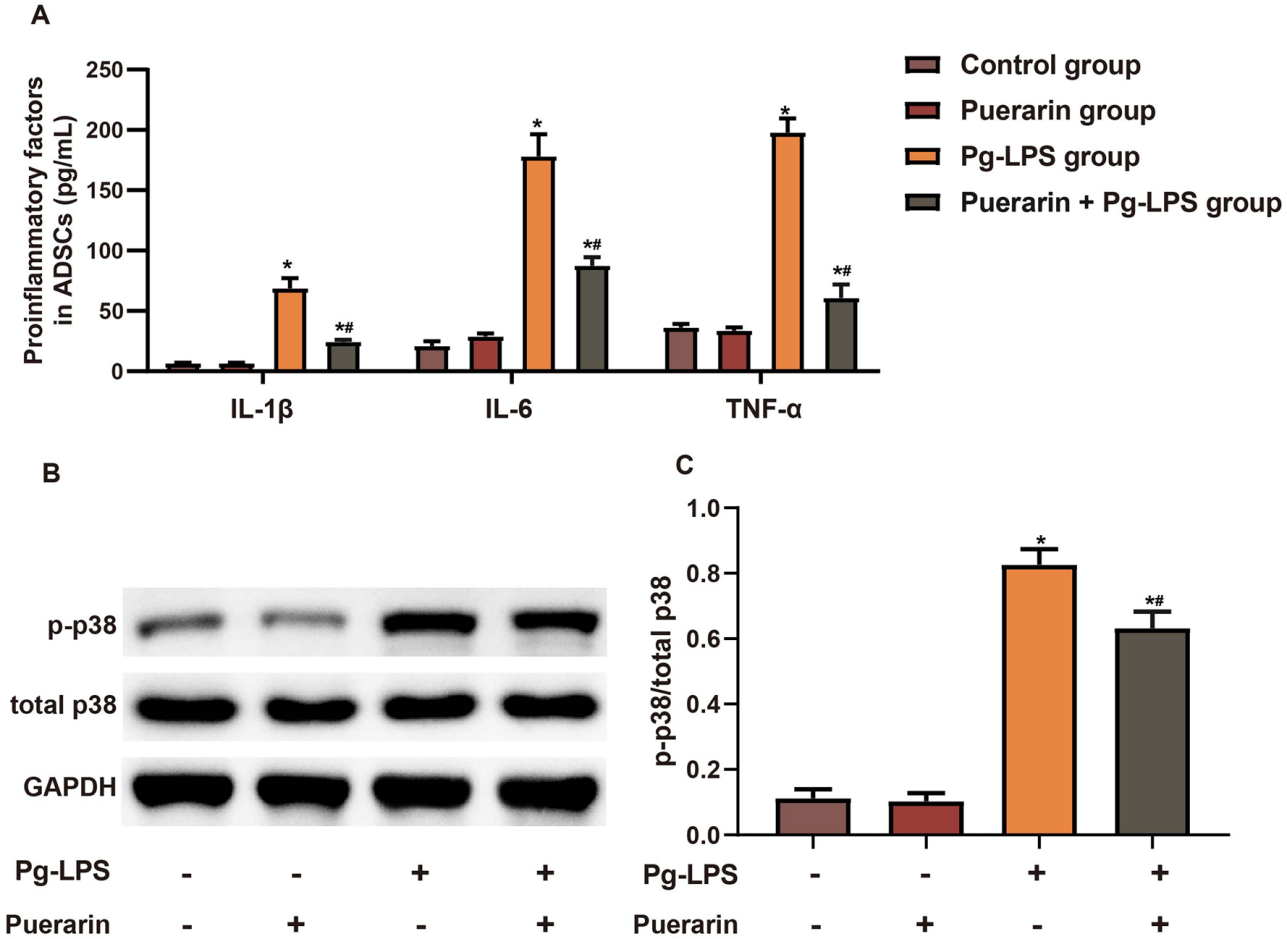
Effect of puerarin on Pg‐LPS induced inflammatory cytokine release and p38MAPK activation in ADSCs. *Note:* (A–C) Levels of pro‐inflammatory cytokines IL‐1β, IL‐6, and TNF‐α in adipose‐derived stem cells (ADSCs) treated with puerarin (10^−6^ M), Pg‐LPS (1 μg/mL), and puerarin + Pg‐LPS, measured by ELISA. (D) Relative phosphorylation levels of p38MAPK (p‐p38/p38 ratio) in ADSCs under the same treatment conditions, assessed by Western blot analysis. Data are presented as mean ± SD. ELISA data represent three independent experiments; Western blotting was performed with *n* = 6 independent biological replicates. Compared to the control group, *p < 0.05; compared to the Pg‐LPS group, #*p* < 0.05.

To assess the effect of puerarin on inflammation induced by Pg‐LPS, the levels of pro‐inflammatory cytokines IL‐1β, IL‐6, and TNF‐α were measured in ADSCs under different treatment conditions (Figure 4A–C). Pg‐LPS treatment significantly increased the secretion of IL‐1β, IL‐6, and TNF‐α compared to the control group (all *p* < 0.05), confirming the inflammatory response. However, puerarin co‐treatment significantly reduced the Pg‐LPS‐induced upregulation of these cytokines (all *p* < 0.05), although their levels remained elevated compared to the control group (all *p* < 0.05). To further investigate the involvement of the p38MAPK pathway, the phosphorylation level of p38 (p‐p38/p38 ratio) was analyzed (Figure 4D). Pg‐LPS treatment resulted in a significant increase in p‐p38/p38 ratio compared to the control group (*p* < 0.05), indicating robust p38MAPK activation. Notably, puerarin co‐treatment significantly reduced Pg‐LPS induced p38 phosphorylation (*p* < 0.05), but the levels remained higher than those in the control or puerarin‐alone groups (*p* < 0.05). These findings suggest that puerarin effectively mitigates Pg‐LPS induced inflammation in ADSCs by suppressing pro‐inflammatory cytokine secretion and inhibiting p38MAPK activation.

### 3.6 Synergistic Effects of ADSCs and Puerarin on Alveolar Bone Preservation and Inflammation Reduction in a Rat Model of Periodontitis

**FIGURE 5:**
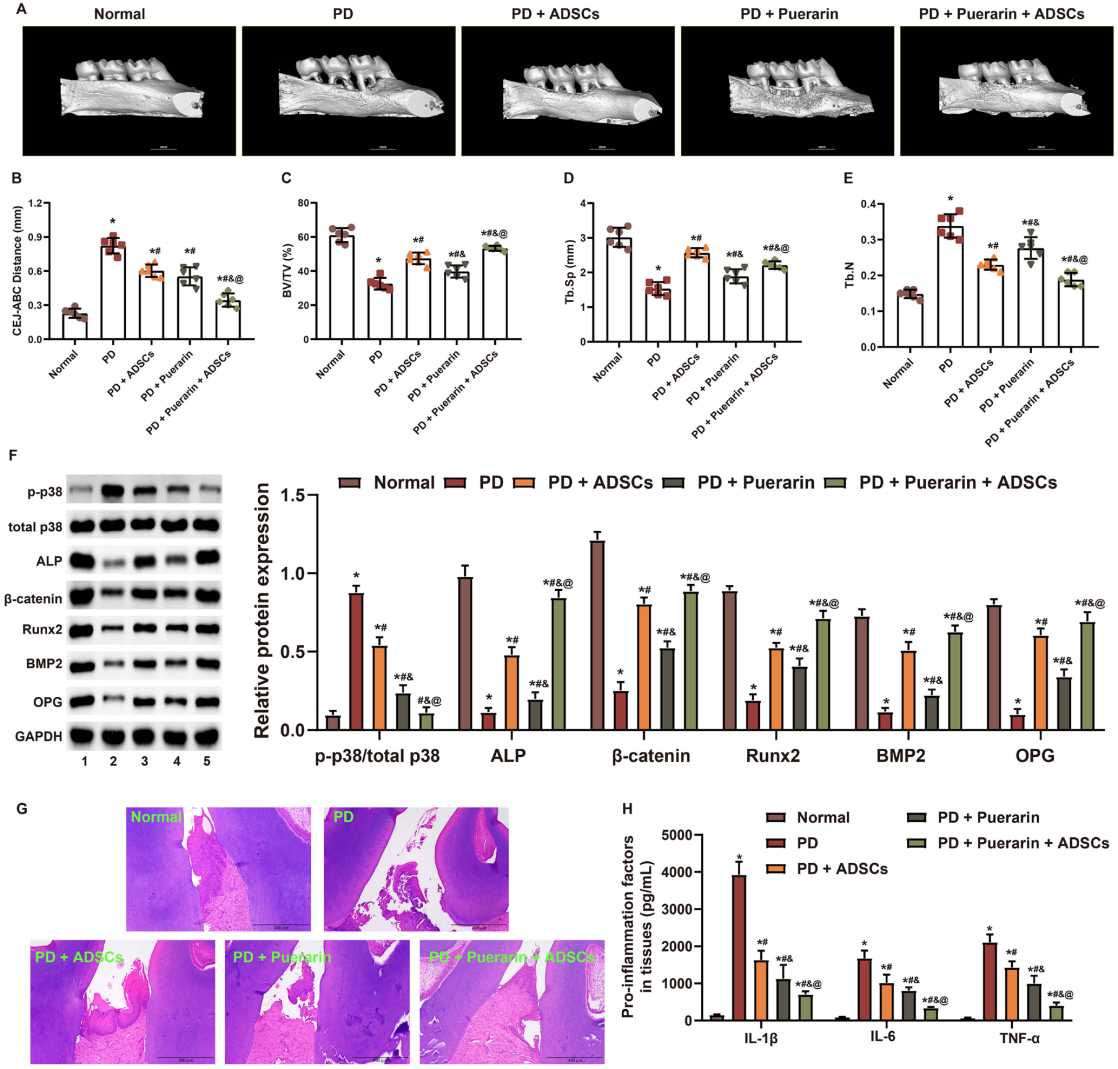
Synergistic effects of ADSCs combined with puerarin on alveolar bone regeneration and inflammation in a rat periodontitis model. *Note:* (A) Representative three‐dimensional micro‐CT reconstructions of the buccal alveolar bone surrounding the maxillary second molar. (B–E) Quantitative micro‐CT assessment of alveolar bone loss and trabecular microarchitecture, including (B) buccal CEJ–ABC distance, (C) bone volume fraction (BV/TV), (D) trabecular separation (Tb.Sp), and (E) trabecular number (Tb.N) across groups. (F) Western blot analysis of p‐p38/p38 signaling and osteogenic markers (ALP, β‐catenin, Runx2, BMP2, OPG) expression in periodontal tissues across different groups. (G) Representative histological images (H&E staining) illustrating periodontal tissue structure and inflammatory infiltration (× 100). (H) ELISA results showing levels of inflammatory cytokines (IL‐1β, IL‐6, TNF‐α) in periodontal tissues. Data presented as mean ± SD (*n* = 6/group). Compared to the normal group, *p < 0.05; compared to the periodontitis (PD) group, #*p* < 0.05; compared to the PD + ADSCs group, &*p* < 0.05; compared to the PD + puerarin group, @*p* < 0.05.

The micro‐CT analysis revealed significant alveolar bone degradation in the periodontitis rats, characterized by increased CEJ‐ABC distance, decreased BV/TV, reduced Tb.N, and elevated Tb.Sp, all statistically significant compared to the normal rats (all *p* < 0.05). Treatments with ADSCs and puerarin ameliorated these changes, with ADSCs showing a more substantial improvement in BV/TV, Tb.Sp, and Tb.N (all *p* < 0.05). However, there was no significant difference in CEJ‐ABC distance reduction between the ADSCs and puerarin groups (*p* > 0.05). The combination of ADSCs and puerarin conferred the most extensive restoration of bone parameters, significantly surpassing each treatment alone, nearly reverting to normal levels (all *p* < 0.05, Figure 5A–E). Western blot analysis indicated that the PD group had heightened p‐p38/p38 ratio, which was markedly reduced by both ADSCs and puerarin treatments, with normalization observed in the combination treatment group (all *p* < 0.05). Additionally, osteogenic markers such as ALP, β‐catenin, Runx2, BMP2, and OPG, diminished in the PD group, were significantly upregulated in the treatment groups, especially pronounced in the combination group (all *p* < 0.05). Notably, ADSCs alone showed a more significant elevation in these markers compared to puerarin alone (all *p* < 0.05, Figure 5F). Histological examination (H&E staining) showed intact periodontal tissue structure with no significant inflammation or bone resorption in the Normal group. In contrast, the PD group exhibited substantial structural damage, extensive inflammatory cell infiltration, and significant alveolar bone resorption. Both PD + ADSCs and PD + puerarin groups displayed improved tissue architecture and reduced inflammation, although bone loss was still apparent. The PD + puerarin + ADSCs group showed the most significant recovery, with markedly reduced inflammation, restored epithelial attachment close to normal levels, and considerably less bone resorption (Figure 5G). Inflammatory cytokine analysis in periodontal tissues further corroborated these findings, showing that IL‐1β, IL‐6, and TNF‐α levels were markedly elevated in the PD group and significantly reduced upon treatment (all *p* < 0.05). The PD + puerarin group, in particular, displayed a more significant decrease in these markers compared to the PD + ADSCs group (all *p* < 0.05), suggesting a potent anti‐inflammatory effect of puerarin. The combination treatment yielded the lowest cytokine levels, suggesting synergistic anti‐inflammatory and tissue‐regenerative effects (all *p* < 0.05, Figure 5H).

## 4 Discussion

In this study, we demonstrated that puerarin exerts significant pro‐osteogenic and anti‐inflammatory effects on ADSCs, ultimately enhancing periodontal tissue repair in a rat periodontitis model. Mechanistically, our data revealed that puerarin at an optimal concentration (10^−6^ M) promotes ADSC proliferation, upregulates key osteogenic markers (ALP, SP7, BSP, and OCN), and facilitates extracellular matrix mineralization. Notably, puerarin reduced the phosphorylation of p38, suggesting that inhibition of p38MAPK signaling plays a central role in its osteogenic and anti‐inflammatory actions. These observations are in line with findings from other studies showing that puerarin can inhibit p38MAPK and alleviate disease‐related conditions [23, 24, 25].

Excessive or sustained activation of the p38 pathway, a major branch of the MAPK cascades typically triggered by stress or inflammatory stimuli [26], is known to compromise osteoblast differentiation by disrupting Runx2 transcriptional activity and interfering with BMP or Wnt/β‐catenin signals [27, 28]. In agreement with Yang et al., who observed puerarin's osteogenic effects in ovariectomized rats, our data indicate that puerarin alleviates these inhibitory signals by reducing p38 phosphorylation [29]. Consequently, it promoted the expression of osteogenic genes such as ALP, SP7, BSP, and OCN, which collectively enhance matrix mineralization.

The capacity of puerarin to protect ADSCs under inflammatory stress provides further mechanistic insight. We showed that LPS stimulation led to marked increases in pro‐inflammatory cytokines (IL‐1β, IL‐6, and TNF‐α), a response closely linked to elevated p38MAPK activity. By inhibiting p38 phosphorylation, puerarin disrupted this inflammatory feedback loop, lowering the expression of those cytokines and creating a more conducive environment for ADSC‐mediated tissue repair [30]. These findings corroborate the work of Liu et al. who reported that puerarin delays LPS‐induced mammary gland aging by inhibiting p38MAPK, thereby reducing senescence‐associated markers and improving mitochondrial function [14]. Consistent with this view, using anisomycin (a p38 activator) reversed puerarin's ability to promote osteogenic differentiation, highlighting that puerarin's efficacy is intimately tied to controlling p38MAPK overactivation.

Our in vivo data showed that co‐administration of puerarin and ADSCs achieved the most favorable therapeutic profile in experimental periodontitis, reflected by improved alveolar bone microarchitecture, reduced local pro‐inflammatory cytokines, and better histological restoration compared with either monotherapy. Notably, ADSCs alone were associated with a relatively greater upregulation of osteogenic‐related proteins (ALP, β‐catenin, Runx2, BMP2, and OPG), whereas puerarin alone produced a more pronounced reduction in inflammatory mediators (IL‐1β, IL‐6, and TNF‐α). Together, these findings support the concept that ADSCs and puerarin exert complementary actions within the periodontal microenvironment. Consistent with previous studies, ADSCs may contribute to periodontal repair through pro‐regenerative and osteoinductive paracrine signaling and/or osteogenic differentiation under permissive conditions [31, 32]. Meanwhile, puerarin appears to provide robust anti‐inflammatory activity, at least in part by dampening p38MAPK overactivation. We speculate that by mitigating the inflammatory milieu and excessive p38MAPK signaling, puerarin may create a more favorable niche that supports ADSC‐mediated tissue repair. This complementary interaction provides a rationale for combined puerarin–ADSC therapy as a candidate strategy for periodontitis management, while acknowledging that direct evidence of ADSC fate in vivo remains to be established.

Several limitations merit consideration. First, although the present work focused on the p38MAPK pathway, anisomycin is also reported to activate JNK [33]. Because JNK signaling was not assessed, pathway specificity cannot be fully resolved. Future studies should quantify phospho‐JNK and incorporate pathway‐selective inhibitors and/or genetic silencing approaches to clarify whether JNK acts independently of or cooperatively with p38MAPK in regulating ADSC function. Second, while the key in vitro Western blot experiments were expanded to six independent biological replicates to improve robustness, other assays were performed with three replicates, which may limit reproducibility; larger sample sizes are warranted in follow‐up work. In addition, the in vivo periodontal tissue Western blot analyses were not further expanded in this revision and should be validated in larger cohorts in future studies. Third, transplanted ADSCs were not tracked in vivo, precluding definitive conclusions regarding their localization, survival/engraftment, or differentiation in periodontal tissues and limiting our ability to disentangle direct osteogenic differentiation from paracrine immunomodulation. Future experiments will incorporate cell‐labeling (e.g., CM‐DiI or GFP) together with ROI‐based co‐localization analyses using osteogenic markers (RUNX2, ALP, OCN/OPN) and immune phenotyping (e.g., iNOS and Arg1/CD206) to define ADSC fate and mechanisms of action. Fourth, the current observation window was sufficient to demonstrate meaningful improvements in bone preservation and inflammatory regulation, but longer‐term follow‐up is required to determine the durability of these effects and to evaluate safety under extended dosing or repeated administration. Finally, a single puerarin dose (10^−6^ M in vitro; 200 mg/kg in vivo) was selected based on preliminary optimization and published studies; systematic dose–response studies and pharmacokinetic/pharmacodynamic evaluations would be important to refine dosing and facilitate future translation.

## 5 Conclusion

In summary, our findings indicate that puerarin harnesses the regenerative capacity of ADSCs by mitigating inflammation and promoting osteogenic differentiation, largely through inhibition of the p38MAPK signaling pathway. When combined with ADSC therapy, puerarin confers synergistic advantages in a rat periodontitis model, as evidenced by improved alveolar bone regeneration and reduced local inflammation.

## Funding

This work was supported by Hunan Provincial Department of Science and Technology (2025JJ80537).

## Conflicts of Interest

The authors declare no conflicts of interest.

## Supporting information


**Figure S1:** Multipotent differentiation potential of adipose‐derived stem cells (ADSCs) Note: (A) Oil Red O staining after 14 days of adipogenic induction, indicating the formation of intracellular lipid droplets. (B) Alcian Blue staining after 21 days of chondrogenic induction, demonstrating increased glycosaminoglycan deposition. (C) Alizarin Red S staining after 21 days of osteogenic induction, confirming the presence of mineralized nodules

## Data Availability

The data that support the findings of this study are available from the corresponding author upon reasonable request.
