# Supplementary figures and images for: Combined Effects of Puerarin and Adipose‐Derived Stem Cells on Alveolar Bone Preservation and Inflammation Control in Periodontitis Through p38MAPK Modulation
Source: Kaohsiung J Med Sci. 2026 Feb 3:e70182. Online ahead of print. doi: 10.1002/kjm2.70182 (PMC13399674; doi:10.1002/kjm2.70182)

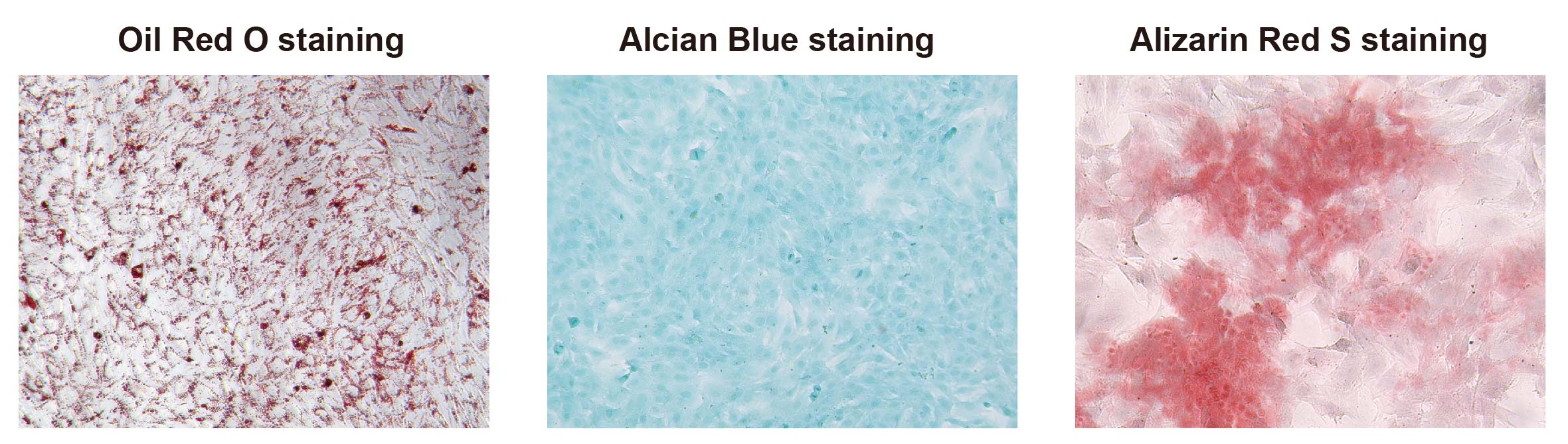

Supplement: Supplementary file 1 — Figure S1: Multipotent differentiation potential of adipose‐derived stem cells (ADSCs) Note: (A) Oil Red O staining after 14 days of adipogenic induction, indicating the formation of intracellular lipid droplets. (B) Alcian Blue staining after 21 days of chondrogenic induction, demonstrating increased glycosaminoglycan deposition. (C) Alizarin Red S staining after 21 days of osteogenic induction, confirming the presence of mineralized nodules [file KJM2-9999-e70182-s001.jpg]
